# Chitosan Glutamate-Coated Niosomes: A Proposal for Nose-to-Brain Delivery

**DOI:** 10.3390/pharmaceutics10020038

**Published:** 2018-03-22

**Authors:** Federica Rinaldi, Patrizia N. Hanieh, Lik King Nicholas Chan, Livia Angeloni, Daniele Passeri, Marco Rossi, Julie Tzu-Wen Wang, Anna Imbriano, Maria Carafa, Carlotta Marianecci

**Affiliations:** 1 Center for Life Nano Science@Sapienza, Istituto Italiano di Tecnologia (ITT), Viale Regina Elena 291, 00161 Rome, Italy; federica.rinaldi@iit.it; 2 Dipartimento di Chimica e Tecnologie del Farmaco, Sapienza Università di Roma, P.zzle A. Moro 5, 00185 Roma, Italy; patrizianadia.hanieh@uniroma1.it (P.N.H.); anna.imbriano@uniroma1.it (A.I.); carlotta.marianecci@uniroma1.it (C.M.); 3 Institute of Pharmaceutical Science, King’s College London, 150 Stamford Street, London SE1 9NH, UK; lik.chan@kcl.ac.uk (L.K.N.C.); julie.tzu-wen.wang@kcl.ac.uk (J.T.-W.W.); 4 Department of Basic and Applied Sciences for Engineering, Sapienza University of Rome, Via A. Scarpa 14, 00161 Rome, Italy; livia.angeloni@uniroma1.it (L.A.); daniele.passeri@uniroma1.it (D.P.); marco.rossi@uniroma1.it (M.R.); 5 Center for Nanotechnology for Engineering (CNIS), Sapienza University of Rome, P. le A. Moro 5, 00185 Rome, Italy

**Keywords:** nose to brain delivery, central nervous system (CNS), chitosan glutamate, niosomes, mucin, pentamidine, dynamic light scattering (DLS), atomic force microscopy (AFM)

## Abstract

The aim of this in vitro study is to prepare and characterize drug free and pentamidine loaded chitosan glutamate coated niosomes for intranasal drug delivery to reach the brain through intranasal delivery. Mucoadhesive properties and stability testing in various environments were evaluated to examine the potential of these formulations to be effective drug delivery vehicles for intranasal delivery to the brain. Samples were prepared using thin film hydration method. Changes in size and ζ-potential of coated and uncoated niosomes with and without loading of pentamidine in various conditions were assessed by dynamic light scattering (DLS), while size and morphology were also studied by atomic force microscopy (AFM). Bilayer properties and mucoadhesive behavior were investigated by fluorescence studies and DLS analyses, respectively. Changes in vesicle size and ζ-potential values were shown after addition of chitosan glutamate to niosomes, and when in contact with mucin solution. In particular, interactions with mucin were observed in both drug free and pentamidine loaded niosomes regardless of the presence of the coating. The characteristics of the proposed systems, such as pentamidine entrapment and mucin interaction, show promising results to deliver pentamidine or other possible drugs to the brain via nasal administration.

## 1. Introduction

Intranasal (IN) drug delivery has been attracting a lot of interest recently due to its potential to bypass hepatic first pass metabolism and the blood–brain barrier (BBB), which is formed by brain endothelial cells with tight junctions, and separates systemic blood circulation and cerebrospinal fluid [1,2,3]. Drugs using other non-invasive delivery methods pass through BBB by paracellular or transcellular pathway, whereas IN delivery has three possible pathways to deliver drugs from nasal cavity to the brain: systemic pathway, olfactory nerve pathway, and trigeminal pathway [3]. For systemic pathway, drug molecules enter the brain by diffusing into blood in the large nasal vesicular network, and then pass through BBB. However, hydrophilic drugs and large molecular weight molecules are inefficiently transported via this pathway, due to the highly selective nature of BBB.

Thus, hydrophilic drugs can be possibly delivered into brain through olfactory and trigeminal nerve pathways. The olfactory nerve cells originate at the central nervous system (CNS) and terminate at surface of olfactory epithelium in the olfactory region, which are located in the roof of nasal cavity. Molecules are transported via the axon, using the paracellular or transcellular route, into olfactory cortex, and then cerebrum and cerebellum. For trigeminal nerve route, it has been proven that drug molecules or nanoparticles diffuse into the maxillary and ophthalmic branches of trigeminal nerve and enter the brainstem [1,2,4]. The major drawbacks of this delivery method are the mucocillary clearance and enzymatic degradation in the nasal cavity, which reduce the drug bioavailability.

One of the challenges of using the intranasal route for drug delivery is its poor bioavailability for hydrophilic drugs, due to its low membrane permeability and rapid mucus clearance system in nasal cavity [5]. Also, several parameters, including pH and temperature, need to be monitored and controlled to simulate conditions in nasal cavity when developing an intranasal drug delivery system.

Niosomes, also called non-ionic surfactant vesicles (NSV), are nanoscale vesicles consisting of an aqueous core and one or multiple lipid bilayers. The vesicles are mainly composed of non-ionic surfactants (e.g., Tween 20) together with cholesterol and charged molecules (e.g., dicetyl phosphate), which are added occasionally to provide greater bilayer stability [6,7,8]. Although only a few niosomal formulations are in clinical trials, and no formulations are in the commercial market, their several advantages, including low toxicity, high chemical stability, ability to entrap hydrophilic or lipophilic drugs, and inexpensive manufacturing cost [8], have provided the unique edge to develop a niosomal medicine product for drug targeting delivery.

Chitosan, a biodegradable and bioadhesive absorption promoter, has been used to overcome the issue of low bioavailability [3,9,10]. It interacts with mucin, which is a major component in nasal mucus layer, and therefore prolongs the drug absorption time via the nasal route. It can also widen the tight junctions between mucosal epithelial cells. Rylomine™, an intranasal chitosan–morphine solution that passed phase III clinical trials, has shown better bioavailability than the solution without chitosan [11]. Moreover, it has also been shown in recent studies that chitosan glutamate (CG) and other chitosan salts have better mucoadhesiveness than chitosan [11,12,13].

There is a renewed interest in pentamidine, an antiprotozoal drug discovered in 1938 for treatment of *Pneumocystis jirovecii* pneumonia, due to the recent discovery of its anti-inflammatory and neuroprotective effect in Alzheimer’s disease (AD) [14,15,16]. It is suggested that pentamidine inhibits S100 calcium-binding protein B (S100B) in glial cells by blocking interaction between S100B and tumor suppressor p53. Overexpression of S100B protein is responsible for upregulation of cell apoptosis and neuroinflammation, which is a key feature of AD [15,16]. The progression of AD could be delayed with anti-inflammatory and antiproliferative effects of pentamidine. However, clinical concerns of pentamidine remain, with its low permeability of the blood–brain barrier and high hepatotoxicity profile.

The aims of this study are to formulate chitosan glutamate-coated niosomal pentamidine for a preliminary characterization, in order to obtain nanocarriers expected to improve pentamidine permeability and reduce side effects. Niosomes with or without chitosan glutamate coating and pentamidine loading have been prepared.

The amount of incorporated polymer is an important aspect in the development of a mucoadhesive formulation and has to be balanced. Low concentrations of polymer (less than 1%) could cause an unstable interaction between polymer and mucus, while high concentrations of polymer (more than 10%) do not necessarily enhance the mucoadhesive properties [17]. The aim of this work is to obtain mucoadhesive coated vesicles characterized by the same physicochemical features of the uncoated ones, by using lower concentrations of polymer. This lower concentration has been chosen to maintain physicochemical features of the coated vesicles comparable with those of the uncoated vesicles, avoiding the overcharging of vesicles in terms of ζ-potential. Thorough physicochemical characterizations have been carried out in terms of the hydrodynamic size, ζ-potential, turbidity, pH, morphology, vesicle bilayer characteristics, mucoadhesion properties, and physical and biological stability, in order to select the formulation most suitable for intranasal administration.

## 2. Materials and Methods

### 2.1. Materials

HEPES salt (Sodium 2-(4-(2-hydroxyethyl)piperazin-1-yl)ethanesulfonate), cholesterol, dicetyl phosphate (DCP), Sephadex G75, pentamidine isethionate, pyrene, 1,6-diphenyl-1,3,5-hexatriene (DPH), mucin from porcine stomach type II powder, Tween 20 (polysorbate 20), chitosan medium molecular weight powder, and sodium hydroxide were purchased from Sigma-Aldrich (Milan, Italy). l-Glutamic acid was supplied by PanReac Applichem (Milan, Italy). All other products and reagents were of analytical grade.

### 2.2. Methods

#### 2.2.1. Niosome Preparation and Purification

Niosomes (Nio) were prepared using thin film hydration method [13,18]. Tween 20 (7.5 mM), cholesterol (15 mM), and DCP (7.5 mM) were dissolved in organic solvent mixture (chloroform/methanol 3:1 *v/v*). The solvent was evaporated using rotary evaporator (VV2000, Heidolph, Schwabach, Germany) to form a thin “film”. The film was hydrated using 5 mL of HEPES buffer (0.01 M pH 7.4) or pentamidine solution (5 mg/mL), vortexed, and sonicated at 60 °C and 16% amplitude for 5 min using ultrasonic microprobe (Vibra-Cell VCX-400, Sonics & Materials, Newtown, CT, USA). The unilamellar vesicle suspension was purified by gel filtration chromatography using Sephadex G75 (glass column of 50 × 1.2 cm) with HEPES buffer as the eluent. The obtained purified vesicles were filtrated with the appropriate pore diameter by using cellulose filters to purify the niosome suspension and obtain desired dimensions.

#### 2.2.2. Preparation of Chitosan Glutamate-Coated Niosomes with and without Pentamidine

Chitosan glutamate (CG) solution was prepared by dissolving C (1 mg) and G (0.82 mg) in acetate buffer (0.2 M, pH 4.4) up to a final concentration of 0.05 mg/mL. The obtained solution was stirred overnight [19].

CG coating of Nio and pentamidine loaded niosomes (NioP) was obtained by adding of CG solution to the different samples into a 1:1 ratio [20]. The obtained suspension was stirred for 1 h at room temperature to achieve CG-coated niosomes (CG-Nio) and CG-coated niosome with pentamidine (CG-NioP).

pH value has been evaluated to confirm for all formulations a suitable pH for nasal administration (3.5 < pH < 6.4) [21,22,23].

#### 2.2.3. Pentamidine Entrapment Efficiency (*EE*%)

Entrapment efficiency (*EE*) of pentamidine in niosomal structure was evaluated by UV–vis spectrometer (Lambda 25, PerkinElmer, Waltham, MA, USA) at 262 nm, and calculated using Equation (1), on samples purified by gel filtration chromatography and filtered on cellulose filters.

(1) $$\mathrm{EE}\;\left(\%\right)\;=\frac{\mathrm{Drug}\;\mathrm{detected}\;\mathrm{in}\;\mathrm{suspension}\;(\mathrm{mg})}{\mathrm{Drug}\;\mathrm{added}\;(\mathrm{mg})}\times 100$$

Obtained results are the average of three different batches ± standard deviation.

#### 2.2.4. Size, ζ-Potential and Morphology of Niosomes

Uncoated niosome (Nio), uncoated niosome with pentamidine (NioP), CG-Nio, CG-NioP, mixture of CG-Nio with mucin, and mixture of CG-NioP with mucin, were characterized. Particle diameter, polydispersity index (PDI), and ζ-potential were measured and analyzed in HEPES buffer by dynamic light scattering (DLS) using a Zetasizer (Nano ZS90, Malvern, UK) (*n* = 3 repeat measurements for each sample) [18].

In order to evaluate the size, the morphology and the homogeneity of the samples, atomic force microscopy (AFM) analysis has been performed using a Dimension Icon (Bruker Inc., Billerica, MA, USA) system. The samples have been prepared by depositing a drop of the niosomes solution after suitable dilution in HEPES on a clean monocrystalline Si (111) wafer. Images (20 μm × 20 μm) of the samples were acquired in standard tapping mode using standard Si probes (RTESP, Bruker Inc., Billerica, MA, USA) in air and at room temperature conditions. The diameter of imaged niosomes was determined by measuring the maximum height (corresponding to the center of the niosome) in respect to the plane of the substrate.

#### 2.2.5. Preparation of Mucin Solution and Mucoadhesive Studies

Mucin powder was dissolved in HEPES buffer to produce a mucin solution (2 mg/mL, pH 6) and stirred overnight at 34 °C [24].

Specific parameters, including temperature (30 °C), concentration of mucin (2 mg/mL), and pH value (6.3–6.7), had been controlled in the mucoadhesive study to mimic the conditions in the nasal mucosal site [23].

Mucin solution (2 mg/mL) was mixed with chitosan glutamate coated niosome (CG-Nio) and pentamidine entrapped chitosan glutamate coated niosome (CG-NioP) suspensions (1:1 ratio), respectively, and incubated at 30 °C [23,25]. The concentration of mucin solution used in this study was modified to obtain optimal pH value (6.3–6.7) [23,26]. Particle size and ζ-potential were measured by DLS (Nano ZS90, Malvern, UK) at 0, 5, 10, and 15 min, to determine the time needed for niosome–mucin complex formation and the stability of the complex. Results reported and discussed related to the data collected at 15 min, even if interaction was almost complete after 5 min, to allow the system to become stabilized.

The interaction between CG-Nio/CG-NioP and mucin were also evaluated by performing fluorescence turbidity measurements using luminescence spectrometer (LS5013, PerkinElmer, Waltham, MA, USA) at Ex/Em 600/600 nm [25] and by AFM studies.

Mucoadhesive measurements were evaluated expressing particle diameter on intensity and number weighted to better appreciate the formation of complex between coated samples with mucin, according to mucoadhesion evaluation performed by Wong et al. [24], in order to avoid artefacts due to the potential mucin aggregates.

#### 2.2.6. Vesicle Bilayer Characterization

Bilayer characterization has been carried out on Nio, NioP, CG-Nio, CG-NioP, and the mixture obtained after adding CG-Nio and CG-NioP with mucin, respectively. Although DPH and pyrene were both lipophilic and located within the bilayer, the probes provided different bilayer information (fluidity, microviscosity, and polarity) due to different fluorescent techniques used to study their behavior inside bilayer. Using both fluorescent probes can provide a wider picture of the characteristics of bilayer as DPH gives an indication of lipid order, while pyrene shows lateral diffusion within the bilayer [27].

Tween 20 (7.5 mM), cholesterol (15 mM), DCP (7.5 mM), and DPH solution (2 × 10^−4^ M) were co-dissolved in chloroform/methanol, which was removed using rotatory evaporator (VV2000, Heidolph, Schwabach, Germany), hydrated in HEPES buffer or pentamidine solution (5 mg/mL), mixed with vortex mixer, and sonicated at 60 °C and 16% amplitude for 5 min. The solution was then filtered through cellulose filter of 450 nm cut off, and its fluorescent measurements were performed (λ = 350–425 nm) using luminescence spectrometer (LS5013, PerkinElmer). The florescence anisotropy (*r*) was determined by using Equation (2) [28,29,30].

(2) $$\mathrm{Florenscence}\;\mathrm{Ansiotropy}\;\left(r\right)=\frac{I_{\mathrm{VV}}{-\mathrm{GI}}_{\mathrm{VH}}}{I_{\mathrm{VV}}{+2\mathrm{GI}}_{\mathrm{VH}}}$$

where $I_{VV}$, $I_{VH}$, $I_{HV}$ and $I_{HH}$ are fluorescent intensities, and subscript *V* (vertical) and *H* (horizontal) represent the orientation of polarized light. *G* factor is ratio of sensitivity of detection system for vertically and horizontally polarized light.

Pyrene loaded niosomes were prepared by adding pyrene (4 mM) with other vesicle components (same preparation method as above). The lateral distribution and the mobility of membrane compounds can be studied by fluorescence measurements. Pyrene is a florescence probe, whose monomer exhibited a spectrum characterized with five emission peaks (from *I_1_* to *I_5_*) and excimer has only one peak (*I_E_*). The monomer and the excimer have different fluorescence signals, and the ratio between the several fluorescence intensities is directly related to the probe distribution in the bilayer. In particular, the ratio *I_1_/I_3_*, corresponding to the first and third vibration bands in the pyrene spectrum, is related to the polarity of the probe environment. Pyrene can form intramolecular excimer based on the viscosity of the probe microenvironment [31], and it is estimated with the ratio *I_E_/I_3_*, where *I_E_* is the excimer intensity. The fluorescence signals emitted by pyrene loaded niosome suspension was scanned (λ = 350–550 nm) using luminescence spectrometer (LS5013, PerkinElmer) and intensities of excimer florescence (*I_E_*), first (*I_1_*), and third (*I_3_*) peak were recorded [32].

#### 2.2.7. Physicochemical Stability

Physical stability studies of CG-Nio and CG-NioP were carried out to investigate if significant size and ζ-potential changes in surfactant vesicle dispersion occur during storage at the two selected temperatures. The vesicular formulations were stored at 4 °C and room temperature for a period of 30 days. Samples from each batch were withdrawn at definite time intervals (1, 8, 15, 22, and 30 days) and the ζ-potential and the mean of hydrodynamic diameter of vesicles were determined as previously described. pH and turbidity changes were also monitored at the beginning and end of the stability test.

Biological studies were also carried out in the presence of artificial cerebrospinal fluid (aCSF) (pH 7.26) to evaluate the stability of vesicular systems after intranasal administration. aCSF was prepared according to MCNay et al. [33]. Mixtures of Nio and 45% aCSF were prepared and incubated at 37 °C. Analyses were performed at different time intervals (0, 0.5, 1, 2, and 3 h) by DLS (Nano ZS90, Malvern, UK) to evaluate variations of particle size and ζ-potential.

Experiments in 45% of aCSF have been also extended up to 24 h, but according to Anderson et al., experiments could last after 3 h [34].

The absorption mechanism of CG-NioP has not been fully investigated. Probably, the chitosan glutamate on the CG-NioP surface should be retained on the olfactory epithelium, and the niosomes or the drug alone should be able to cross the nasal mucosa. For this reason, only the uncoated NioP stability in aCSF was determined [3].

#### 2.2.8. In Vitro Release Studies

The pentamidine release by CG-Nio was tested for in vitro release experiments. The experiments were carried out using dialysis tubes (molecular weight cutoff 8000 and 5.5 cm^2^ diffusing area) at 37 °C in HEPES buffer (10 mM, pH 7.4) or aCSF. The set-up was kept at T = 37 °C by means of a temperature-controlled water bath, and the release medium was gently magnetically stirred during the experiment.

Aliquots of 1 mL were withdrawn from the solution at appropriate time intervals to perform UV analyses and then re-inserted back in the external medium. Released pentamidine was detected by means of a spectrophotometer (Perkin-Elmer, lambda 3a, UV–vis spectrometer), as described above.

All aliquots were analyzed immediately after sampling. All release experiments were carried out in triplicate. The values reported in the present paper represent the mean values, and lay within 10% of the mean.

#### 2.2.9. Statistical Analysis

Results are expressed as the mean of three independent experiments ± standard deviation. The data were statistically analyzed using Minitab-18 and Excel for Mac 2011. Significance of data was performed using one-way analysis of variance (ANOVA), and differences were significantly different when *p* < 0.05.

## 3. Results and Discussion

### 3.1. Characterization of Niosomes

The niosome samples were tested for their size, ζ-potential, and PDI using dynamic light scattering analyses. From the obtained data showed in Table 1, the addition of chitosan glutamate lead to the coating of niosomes with an increase in ζ-potential values to less negative ones [18]. The increase in ζ-potential is due to electrostatic interaction between positive charged chitosan and negatively charged DCP on the surface of niosome bilayer [35]. Although the size of CG-Nio is relatively small (~120 nm), it is not small enough for nose to brain delivery via intra-axonal route, which needs a diameter of 100 nm [2]. It is therefore likely that the formulated CG-niosome will be transported into the brain through different pathway; however, this hypothesis will need to be confirmed with further in vivo studies.

Nanocarrier size is a crucial parameter for penetration within the brain (regardless of administration route) and cell uptake: Nance et al. [36] estimated that that human brain tissue extracellular space has more than one-quarter of all pores ≥100 nm. These findings were confirmed in vivo in mice, where 40 and 100 nm nanoparticles spread rapidly within brain tissue, only if densely coated with the hydrophilic polymer (PEG), and Tween 20 bears 20 PEG units on its polar head.

It is a real challenge, when verifying, to assess the efficacy of nanocarriers as useful tools to nose to brain delivery [37]. Recently, the nose-to-brain route has been receiving ever-increasing interest, as shown by about 800 research articles published in the last three years (data retrieved from Scopus).

Characterization studies on different coating polymer concentration, and its effect on size and ζ-potential values, have been carried out. Obtained data are summarized in Table 1.

The CG concentration range used, after dilution in the niosomal suspension, was fixed between 0.05 mg/mL and 1 mg/mL. The coating with cationic CG was demonstrated by the increase to less negative values due to the electrostatic attraction between negative niosomes and positive CG. The CG concentration of 0.05 mg/mL was selected for further characterization, since, at this polymer concentration, the *Z*-average remained under 150 nm and the PDI value was still acceptable. Furthermore, a ζ-potential of −25 mV confirmed the niosome coating and prevented niosome aggregation over time [38].

As reported in Table 2, an increase of vesicle dimension was observed when pentamidine was added to the formulation by comparing the dimension between Nio and NioP. When CG was added to Nio and NioP, no significant increase in dimension occurred. The increase of ζ-potential was slightly smaller than drug free niosome as the pentamidine is embedded in the bilayer, and may have affected the electrostatic interaction between chitosan and bilayer.

CG-Nio and CG-NioP were larger than 100 nm, and it can be presumed that they will be mainly transported via trigeminal nerve pathway. All samples had a PDI value higher than 0.2, so they cannot be considered as monodisperse suspensions. A study using low molecular weight chitosan has recorded significant rise in CG-Nio size when chitosan was added [18].

**Figure 1:**
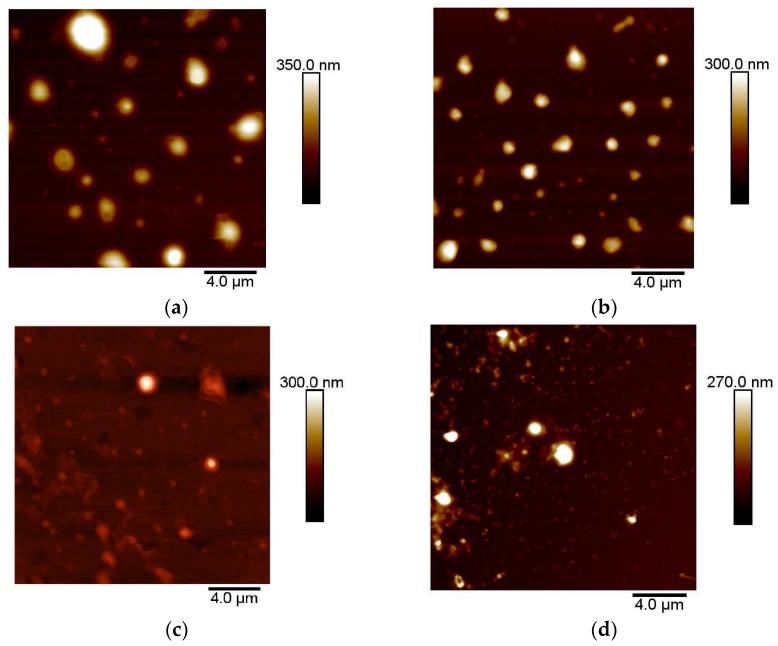
Microscopic images using AFM. (**a**) uncoated niosomes (Nio); (**b**) uncoated niosomes with pentamidine (NioP); (**c**) chitosan glutamate coated niosomes (CG-Nio); (**d**) chitosan glutamate coated niosomes with pentamidine (CG-NioP).

Typical topographical images of Nio, NioP, CG-Nio, and CG-NioP were shown in Figure 1a–d, respectively, and confirmed their spherical shape, their relatively not so homogeneous and non-coalescence size distribution. The vesicle mean diameters determined from AFM images (Table 2) are comparable with those obtained by DLS analysis, which is actually more precise because the number of analyzed particle by AFM is much smaller. Furthermore, it should be considered that the approaches of the two methods are different, but anyhow, the obtained data are within the same range.

The Entrapment efficiency for NioP was 10.96% (concentration of pentamidine in niosomal suspension is 0.548 mg/mL). This concentration is useful to achieve therapeutic efficacy [15].

### 3.2. Physicochemical Stability

Vesicle stability is a complex issue and involves chemical stability, physical stability, and biological stability, which are all interrelated. The evaluation of these parameters is fundamental to determine the potential in vitro/in vivo applications in nanomedicine. Generally, stability is determined by means of size and ζ-potential variations (DLS, Turbiscan Lab Expert or microscopy techniques) or by evaluation of the release rate of different probes as a function of time and/or temperature, in the absence or presence of biological fluids [39].

**Figure 2:**
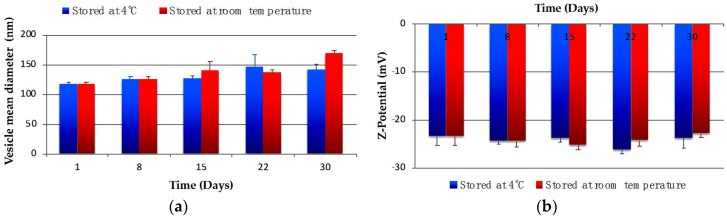
Changes of CG-Nio in (**a**) hydrodynamic diameter and (**b**) ζ-potential within 30 days at different storage temperatures. Values represent the mean ± standard deviation of *n* = 3 niosome sample measurements.

**Figure 3:**
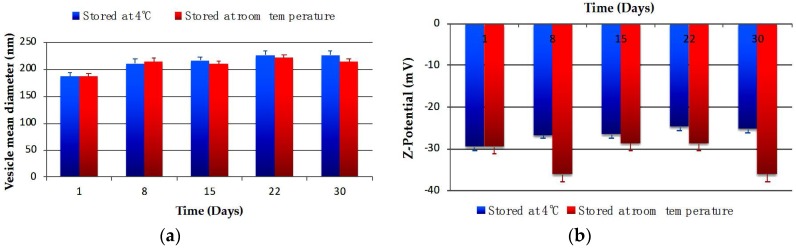
Changes of CG-NioP in (**a**) hydrodynamic diameter and (**b**) ζ-potential within 30 days at different storage temperatures. Values represent the mean ± standard deviation of *n* = 3 niosome sample measurements.

The physical stability studies of CG-Nio and CG-NioP are shown in Figure 2 and Figure 3.

The mean vesicle size for CG-Nio stored at 4 °C and at room temperature, shown in Figure 2a, does not show a significant change in 30 days (*p* > 0.05). The ζ-potential values for drug free niosomes stored at 4 °C and room temperature shown in Figure 2b had no significant differences (*p* > 0.05) at the beginning and end of 1-month period.

For pentamidine entrapped niosomes, the size, reported in Figure 3a, shows a slightly increase after 7 days, but remained stable in the period of 1 month at both storage temperatures. As shown in Figure 3b, fluctuations in ζ-potential of the samples stored at room temperature, were observed in the one-month period, while no variations were observed for samples stored at 4 °C. PDI for CG-NioP, as both storage temperatures were below 0.4, which indicated the suspension remained as monodisperse system (data not shown) [40,41].

### 3.3. Vesicle Bilayer Characterization

The effects of chitosan glutamate on niosome bilayer were investigated using fluorescent probes, and the data are shown in Table 3. Both CG-Nio and CG-NioP had similar fluorescence anisotropy values compared to Nio and Nio-P respectively, indicating chitosan glutamate has no/little effect on bilayer fluidity. As fluorescence anisotropy values were inversely correlated to bilayer fluidity [32], the low values in NioP and CG-NioP suggested that the presence of the drug gives a more fluid (lateral movement within membrane) bilayer than drug free niosomes [42].

Polarity values of vesicle bilayer were similar between Nio, CG-Nio empty and pentamidine loaded. This indicates the addition of chitosan glutamate onto niosome had no effect on the bilayer polarity.

From Table 3, it can be observed that the microviscosity values for samples with pentamidine were much lower than the samples without drugs, regardless of the presence of chitosan glutamate. This data may indicate that pentamidine molecule may have interrupted the bilayer structure and lowered the viscosity. The presence of pentamidine in niosome had decreased the bilayer microviscosity, due to their ability to insert into the bilayer and compress the apolar region of the bilayer with a consequent increase of rigidity.

### 3.4. Mucoadhesive Study

Interactions between mucin (main component in mucus) and coated/uncoated niosomes were evaluated using DLS to determine the differences in size and surface charge before and after addition of mucin. The size, ζ-potential, pH, and turbidity values listed in Table 4, Table 5 and Table 6 all confirmed the interaction of mucin with coated niosome, as incubated mixtures have values between the mucin and niosome sample alone (no evidences of mucin interaction were collected for uncoated samples, data not shown).

Although the negative ζ-potential values of coated niosomes, the mucin interaction occurs probably due to a “non-specific” mucin interaction and a physical entanglement between the polymer and mucosal layer [43].

**Figure 4:**
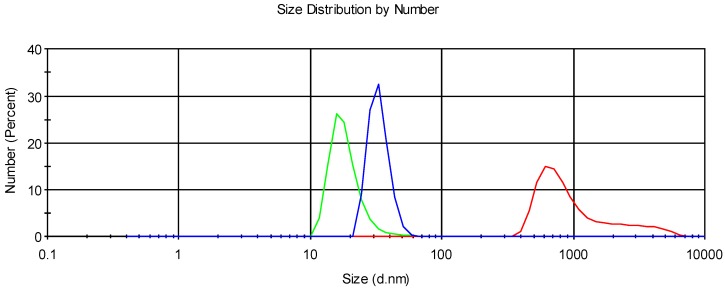
Particle number weighted size distribution of Nio (green), Nio–mucin mixture (blue), and mucin solution (red).

**Figure 5:**
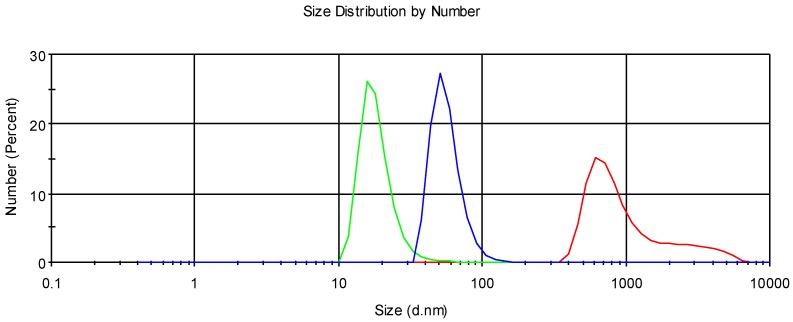
Particle number weighted size distribution of CG-Nio (green), CG-Nio–mucin mixture (blue), and mucin solution (red).

**Figure 6:**
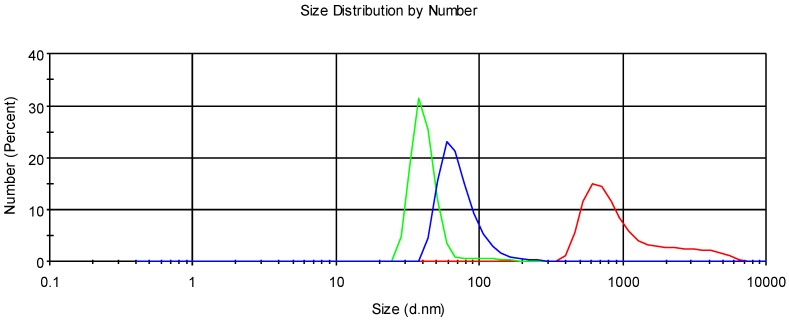
Particle number weighted size distribution of CG-NioP (green), CG-NioP–mucin mixture (blue), and mucin solution (red).

The PDI values of the mixtures were significantly higher than niosome sample alone, due to the polydisperse system of the mixtures. Similar trends were also observed with mixture of mucin and pectin-coated liposomes, where pectin has similar properties as chitosan glutamate [25]. Particle number weighted size distribution curves for mucin alone, mucin–niosome mixture, and different niosome samples were illustrated in Figure 4, Figure 5 and Figure 6, and certain degrees of interaction between niosomes and mucin were shown in the three distribution graphs (Nio, CG-Nio, and CG-NioP). The sizes listed in Table 4, Table 5 and Table 6 are different to the size dimensions in Figure 4, Figure 5 and Figure 6. The number distribution curves (Figure 4, Figure 5 and Figure 6) show the number of particles in the different sizes, while the sizes in Table 4, Table 5 and Table 6 were obtained using intensity distribution, which describes amount of light scattered by particle sizes. The bell-shaped size distribution curves of niosome–mucin mixture were located between the curves of mucin alone and niosome alone, which are a result of the adhesive properties of uncoated and coated niosome samples, and suggest the successful formation of the complexes. However, further experiments, including mucous glycoprotein assay, which determines the free mucin concentration in mixtures, should be done to investigate the full extent of rise in mucoadhesive properties after the addition of chitosan glutamate into niosomes.

In Table 4, Table 5 and Table 6 variation in pH and turbidity are also reported for the three different samples in absence or presence of mucin. pH values are always compatible with those expected for a nasal administration. Turbidity values, after addition of mucin, assume intermediate values between niosomes and mucin alone, confirming the interaction of the two dispersions.

From data obtained by mucoadhesive studies, it can be highlighted that the mucin interaction occurs both in uncoated and in coated niosomes. This is probably related to the presence of PEG units in Tween 20 molecules, and consequently, on niosomal surface. PEG itself, in fact, shows mucoadhesive properties [44]. Furthermore, the addition of CG and its presence on niosomal surface is fundamental because of its penetration enhancer properties [45].

**Figure 7:**
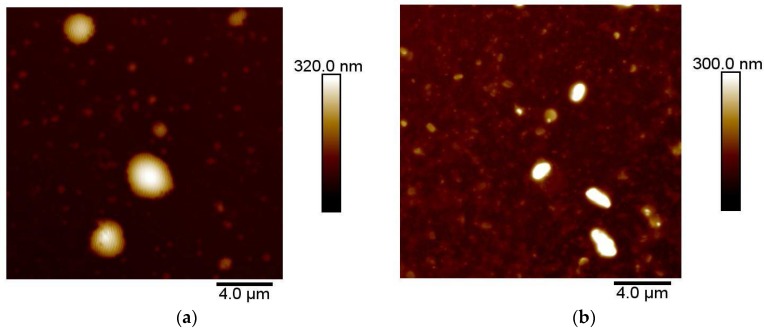
Microscopic images using AFM. (**a**) CG-Nio with mucin, and (**b**) CG-NioP with mucin.

Sample CG-Nio in the presence of mucin does not change its morphology, while CG-NioP with mucin (Figure 7a,b) showed elongated niosomes and a change in morphology in the presence of mucin. The observed dimensions of all samples were in agreement with the ones obtained by DLS analyses showed above. 

### 3.5. Stability in Artificial Cerebrospinal Fluid

**Figure 8:**
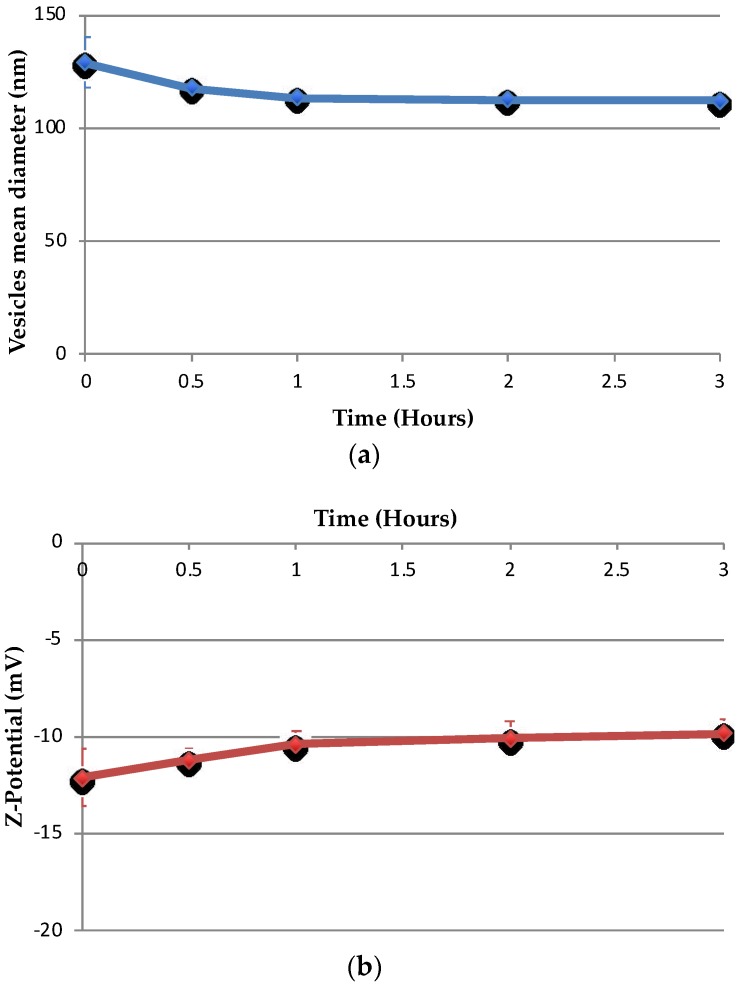
Changes of Nio–aCSF mixture in (**a**) hydrodynamic diameter and (**b**) ζ-potential over a 3 h period. Values represent the mean ± standard deviation of *n* = 3 sample measurements.

**Figure 9:**
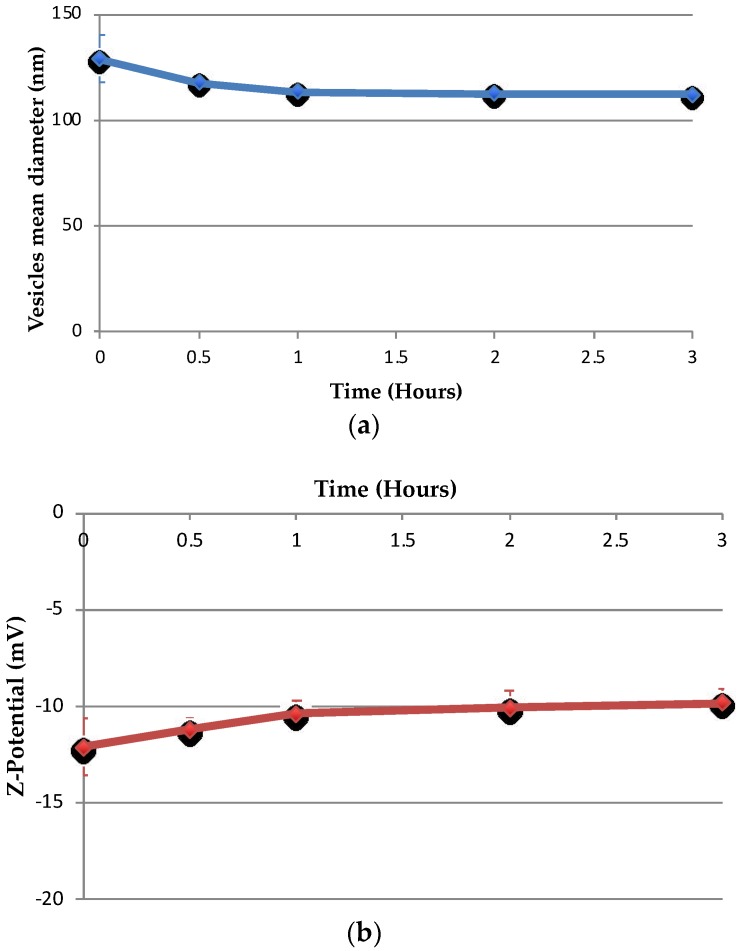
Changes of NioP–aCSF mixture in (**a**) hydrodynamic diameter and (**b**) ζ-potential over a 3 h period. Values represent the mean ± standard deviation of *n* = 3 sample measurements.

The changes in size and ζ-potential for drug-free and drug-loaded niosomes are shown in Figure 8 and Figure 9, respectively. Nio had no statistically significant changes in size within 3 h after addition of aCSF (*p* = 0.066), whereas NioP had statistically significant changes in size (*p* = 0.036) and no significant changes in ζ-potential for both samples after aCSF was added (*p* = 0.076 and *p* = 0.04 respectively). Release profile of liposomal bupivacaine, a hydrophilic drug, in aCSF, was investigated by Düzlü AÜ et al. [46]. It is suggested that the drug release in aCSF was slow and controlled, due to the existence of lipid bilayer in liposome and re-dispersion of liposomes in aCSF. From the results in Figure 8 and Figure 9, it can be confirmed that niosome vesicles remain stable and would not burst/shrink in a short period of time when contacting with cerebrospinal fluid. As the structural similarities between liposome and noisome, it can only be theoretically hypothesized that the drug-free and pentamidine-containing niosomes were separated and re-dispersed in aCSF, which leads to the slight changes in niosomes size, whereas the ζ-potential remain constant. Further experiments, including in vitro release studies, must be done to confirm the hypothesis.

### 3.6. Pentamidine Release Studies

**Figure 10:**
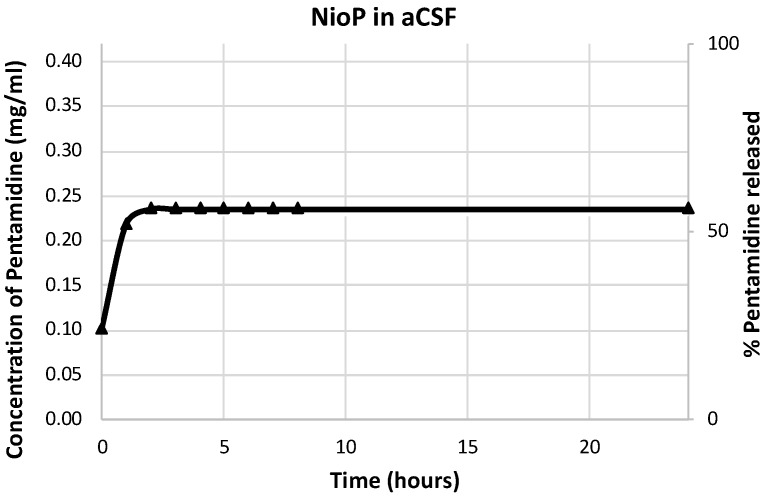
release profile of pentamidine by CG-Nio expressed as concentration or percentage of pentamidine released.

Release profiles of pentamidine by CG-Nio in aCSF are shown in Figure 10. From the obtained results, it is possible to affirm that 50% of pentamidine present in the formulation is released within 24 h. This percentage corresponds to the 50% of pentamidine entrapped in NioP suspension in presence of aCSF 1:1 (*v/v*). In particular, 50% of release is reached in 3 h, and this must be due to pentamidine release by entire vesicles because vesicle stability in aCSF was already assessed. This amount of release is useful to achieve a pharmacological effect [14].

## 4. Conclusions

In conclusion, both CG-Nio and CG-NioP obtained from thin film hydration method seem to show in vitro mucoadhesive properties. However, further studies are needed to verify, in vivo, the improved bioavailability and the reduced drug clearance in nasal cavity.

## Figures and Tables

**Table 1:** Niosomal dimensions (nm) and ζ-potential (mV) values obtained by adding of chitosan glutamate (CG) solution to the free niosomal samples in a 1:1 ratio.

Sample	Hydrodynamic Diameter (nm) ± SD	ζ-Potential (mV) ± SD	PDI ± SD
Nio	145.4 ± 2.5	−44.2 ± 2.2	0.346 ± 0.04
CG-Nio (0.02 mg/mL)	121.7 ± 1.0	−35.8 ± 0.8	0.390 ± 0.01
CG-Nio (0.05 mg/mL)	117.9 ± 2.4	−26.7 ± 0.7	0.404 ± 0.04
CG-Nio (0.50 mg/mL)	314.0 ± 3.3	−14.6 ± 0.7	0.510 ± 0.02
CG-Nio (1.00 mg/mL)	1000.0 ± 0.1	-	0.980 ± 0.01

**Table 2:** DLS and AFM analyses on niosomes in presence and absence of pentamidine and chitosan glutamate. * Values represent the mean ± standard deviation of *n* = 3 niosome sample measurements. ** Values represent the mean ± standard deviation calculated on at least 15 different niosomes.

Sample	Hydrodynamic Diameter (nm) *	ζ-Potential (mV) *	PDI *	Vesicles Mean Diameter (nm) from AFM **
Nio	126.3 (±0.8)	−44.3 (±1.4)	0.363 (±0.044)	177 (±15)
CG-Nio	117.9 (±2.4)	−23.4 (±1.9)	0.347 (±0.002)	144 (±10)
NioP	165.2 (±3.1)	−41.6 (±1.4)	0.211 (±0.020)	179 (±15)
CG-NioP	180.2 (±1.5)	−29.5 (±1.6)	0.248 (±0.016)	182 (±17)

**Table 3:** Uncoated and CG coated Nio and NioP bilayer characteristics.

Sample	Fluidity (Anisotropy)	Microviscosity (*I_E_/I_3_*)	Polarity (*I_1_/I_3_*)
Nio	0.29	0.75	1.08
CG-Nio	0.29	0.86	1.05
NioP	0.19	0.27	1.19
CG-NioP	0.15	0.24	1.18

**Table 4:** Parameter measurements after Nio contacted with mucin.

Sample	Hydrodynamic Diameter (nm)	ζ-Potential (mV)	PDI	pH	Turbidity
Nio	145.4 (±2.5)	−44.2 (±2.2)	0.346 (±0.04)	7.4	196.0
Nio–mucin	238.0 (±15.1)	−20.4 (±1.3)	0.762 (±0.10)	7.1	224.0
Mucin	1623.0 (±57.0)	−15.6 (±0.4)	0.452 (±0.08)	6.1	257.3

**Table 5:** Characteristics of CG-Nio contacted with mucin dispersion.

Sample	Hydrodynamic Diameter (nm)	ζ-Potential (mV)	PDI	pH	Turbidity	Mean Diamter from AFM (nm)
CG-Nio	117.9 (±2.4)	−26.7 (±0.7)	0.404 (±0.04)	5.0	98.0	144 (±10)
CG-Nio–mucin	255.0 (±7.1)	−20.4 (±1.3)	0.878 (±0.05)	5.5	172.6	213 (±18)
Mucin	1623.0 (±57.0)	−15.6 (±0.4)	0.452 (±0.08)	6.1	257.3	-

**Table 6:** Parameter measurements after CG-NioP contacted with mucin.

Sample	Hydrodynamic Diameter (nm)	ζ-Potential (mV)	PDI	pH	Turbidity	Mean Diameter from AFM (nm)
CG-NioP	165.1 (±3.2)	−26.6 (±1.3)	0.158 (±0.03)	4.7	623.4	182 (±17)
CG-NioP–mucin	200.3 (±9.6)	−18.7 (±0.5)	0.349 (±0.03)	5.3	435.6	218 (±16)
Mucin	1623.0 (±57.0)	−15.6 (±0.4)	0.452 (±0.08)	6.1	257.3	-
